# Epidemiology, clinical characteristics, resistance, and treatment of infections by *Candida auris*

**DOI:** 10.1186/s40560-018-0342-4

**Published:** 2018-10-29

**Authors:** Andrea Cortegiani, Giovanni Misseri, Teresa Fasciana, Anna Giammanco, Antonino Giarratano, Anuradha Chowdhary

**Affiliations:** 1 Department of Surgical, Oncological and Oral Science (Di.Chir.On.S.). Section of Anesthesia, Analgesia, Intensive Care and Emergency. Policlinico Paolo Giaccone. University of Palermo, Italy, University of Palermo, Via del vespro 129, 90127 Palermo, Italy; 2 Department of Sciences for Health Promotion and Mother and Child Care, University of Palermo, Palermo, Italy; 3 Department of Medical Mycology, Vallabhbhai Patel Chest Institute, University of Delhi, Delhi, India

**Keywords:** *C. auris*, *Candida*, Candidemia, Invasive fungal infection, Antimicrobial resistance, Antifungal resistance

## Abstract

*Candida* spp*.* infections are a major cause of morbidity and mortality in critically ill patients. *Candida auris* is an emerging multi-drug-resistant fungus that is rapidly spreading worldwide. Since the first reports in 2009, many isolates across five continents have been identified as agents of hospital-associated infections. Independent and simultaneous outbreaks of *C. auris* are becoming a major concern for healthcare and scientific community. Moreover, laboratory misidentification and multi-drug-resistant profiles, rarely observed for other non-albicans *Candida* species, result in difficult eradication and frequent therapeutic failures of *C. auris* infections. The aim of this review was to provide an updated and comprehensive report of the global spread of *C. auris*, focusing on clinical and microbiological characteristics, mechanisms of virulence and antifungal resistance, and efficacy of available control, preventive, and therapeutic strategies.

**Electronic supplementary material:**

The online version of this article (10.1186/s40560-018-0342-4) contains supplementary material, which is available to authorized users.

## Introduction

*Candida* spp*.* infections are a major cause of morbidity and mortality in critically ill patients [1–3]. Yeasts of genus *Candida* are associated with a wide range of different clinical manifestations, including bloodstream infections (BSIs), intra-abdominal candidiasis, deep-seated candidiasis, and superficial infections [1, 4, 5]. Infections caused by *Candida* spp. have progressively increased over the last decades, and this phenomenon is mainly associated with the increasing rate of invasive procedures, the extensive use of broad-spectrum antimicrobials, and the more frequent immunocompromised status of critically ill patients [6–8]. Although *Candida albicans* still remains the main agent of hospital-acquired fungal infection, several species of non-albicans *Candida* namely *C. tropicalis*, *C. glabrata*, *C. parapsilosis*, and *C. krusei* account for increasing incidence of invasive infections with high rates of therapeutic failure, mainly related to echinocandins and azoles resistance [9–11]. Current increase in antifungal drug resistance is not only linked to the acquired mechanism following administration of antifungal agents but intrinsic resistance to several classes of antimicrobials among different non-*albicans* species has also been recorded [12].

*C. auris* is an emerging multi-drug-resistant fungus that is rapidly spreading worldwide. Since the first reports in 2009, many isolates have been identified across five continents as agents of hospital-associated infections [11, 13, 14]. Reported cases are characterized by high overall mortality [15, 16] and high rate of antifungal resistance [17]. Of note, most reported infections involved critically ill patients [15, 18]. Moreover, difficulty in microbiological identification [19, 20], high virulence [21–23], multi-drug resistance profile [24, 25], and rapid global spread with several reported outbreaks ([11, 26, 27]; (https://www.cdc.gov/fungal/diseases/candidiasis/tracking-c-auris.html); [28]) lead the healthcare and scientific communities to consider *C. auris* as one of the most serious emerging pathogen that critical care physicians should be aware of.

The aim of this review is to provide an updated report of the global spread of *C. auris* focusing on clinical and microbiological characteristics, mechanisms of virulence and antifungal resistance, and efficacy of available control, preventive, and therapeutic strategies.

## Main text

### Systematic review

For the purpose of this review, we performed a systematic review of the literature using “Candida” AND “auris” as keywords. We searched the PubMed, Scopus, and Web of Science. We excluded articles in languages other than English. Two authors (A. C. and G. M.) independently performed the search. Differences in selections were solved by consensus, with the help of the third author (T. F.). We included peer-review articles and meeting abstracts, concerning epidemiology, clinical manifestations and risk factors, virulence, genotypic characteristics, and therapeutic management. Concerning clinical cases, we included all cases of isolation of *C. auris* in humans reported in literature. Cases were defined as patients in whom *C. auris* was isolated, and this definition includes both superficial and deep-seated infections. We also checked references of relevant articles to find potential articles not retrieved by the databases search. After excluding not relevant articles and duplicates, we included 131 relevant articles published from 2009 to 30 May 2018. Articles retrieved were further categorized as shown in the flow diagram, following PRISMA guidelines (Additional file 1).

### Microbiological characteristics of *C. auris*

**Fig. 1:**
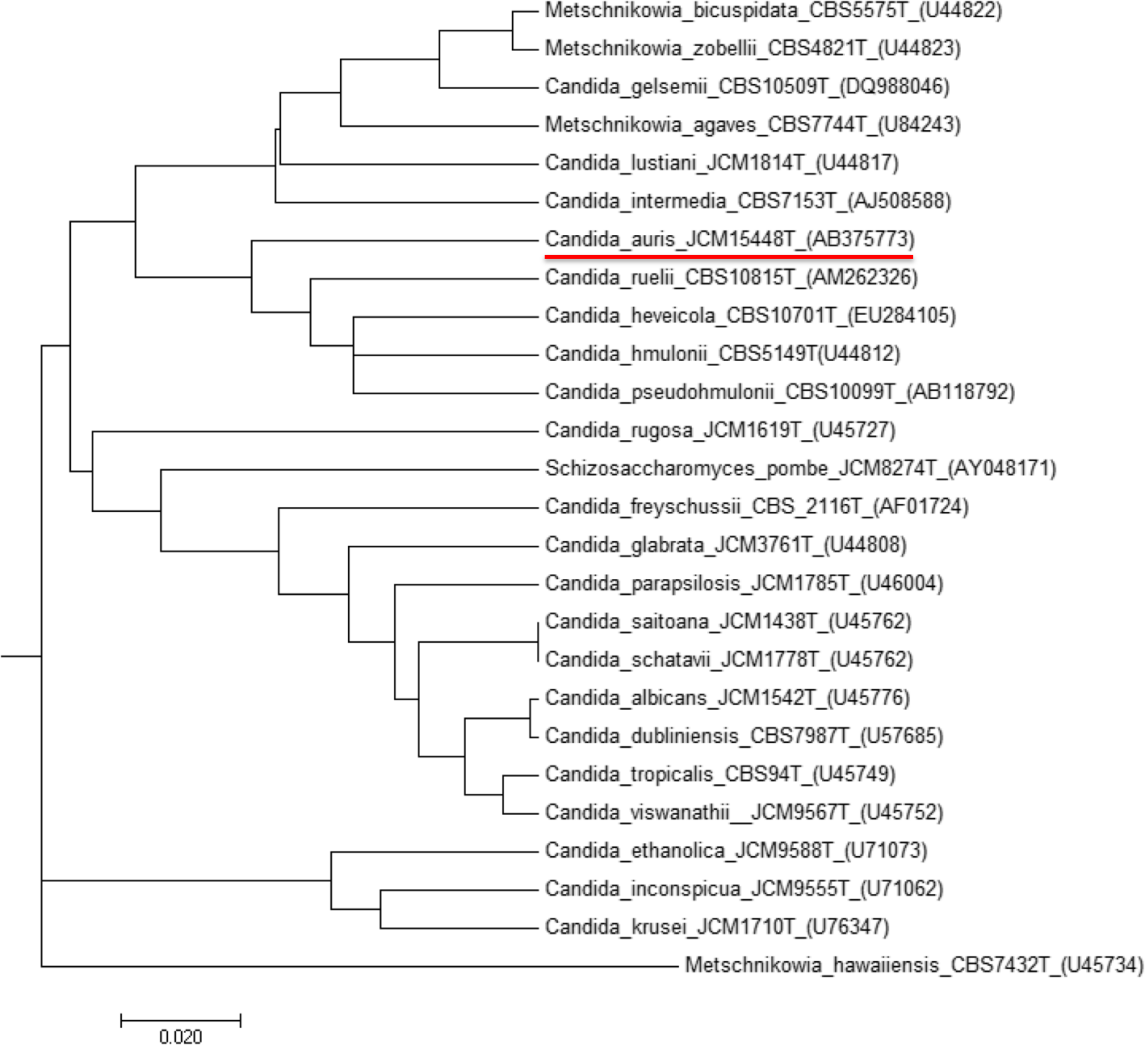
Phylogenetic tree obtained by neighbor-joining analysis of the D1-D2 region of genes encoding Candida auris 26S rRNA and correlated species

On Sabouraud’s agar, *C. auris* produces smooth and white cream-colored colonies, which are germ tube test negative. On CHROMagar *Candida* medium, *C. auris* produces colonies that may appear pale to dark pink, or rarely beige. The yeast *C. auris* is able to grow at 42 °C, and this characteristic helps differentiate *C. auris* from *C. haemulonii*, which does not grow at these temperatures [19]. The microscopic morphology of *C. auris* cells appears to be oval without pseudohyphae formation. However, *C. auris* might exhibit multiple morphological phenotypes under different cultures conditions, including round-to-ovoid, elongated, and pseudohyphal-like forms. For instance, high concentrations of sodium chloride induce the formation of a pseudohyphal-like form [29]. Cycloheximide 0.1% and 0.01% inhibits its growth [30]. The phenotypic, chemotaxonomic, and phylogenetic characteristics (Fig. 1) have therefore clearly suggested that it was a new species affiliated to the genus *Candida* (anamorphic) and therefore to the class of *Ascomycetes* even if the perfect form is not known (teleomorphic). Whole genome phylogeny of *C. auris*, *C. haemulonii*, *C. duobushaemulonii*, and *C. pseudohaemulonii* showed that they represent a single clade, confirming the close relationship of these species [31]. Due to the close genetic relatedness with *C. haemulonii* complex, *C. auris* is often commonly misidentified as *C. haemulonii* in routine diagnostic laboratories using biochemical methods. In fact, commercially available biochemical-based tests, including API AUX 20C, VITEK-2 YST, BD Phoenix, and MicroScan, misidentify *C. auris* as a wide range of *Candida* species and other genera. Misidentifications yielding *C. famata, C. sake*, *Rhodotorula glutinis*, *Rhodotorula mucilaginosa*, *Saccharomyces*, *C. catenulate*, *C. lusitaniae*, *C. guilliermondii*, and *C. parapsilosis* have been reported [19, 20, 26]. Recently, BioMerieux has updated the database [32, 33] and inclusion of *C. auris* spectra in the VITEK-2 system yields to its correct identification. Matrix-assisted laser desorption/ionization time-of-flight (MALDI-TOF) mass spectrometry can reliably differentiate *C. auris* from other *Candida* species, provided *C. auris* spectrum is included in the reference database and by selecting appropriate extraction method [34, 35]. The development of specific PCR assays for *C. auris* and for *C. auris*-related species using cultured colonies seems promising for its rapid and accurate identification, particularly in outbreak settings [36, 37]. Molecular identification of *C. auris* can be performed by sequencing various genetic loci (including *D1/D2*, *RPB1*, *RPB2*, and internal transcribed spacer *ITS1*, *ITS2*), but it is not routinely used [38, 39].

### Epidemiology trends and world outbreaks

The real prevalence and the epidemiology of *C. auris* still remain uncertain. One of the causes may be the underestimation of its isolation due to the limited accuracy of available conventional diagnostic tools [40]. With the purpose to investigate whether *C. auris* emerged in recent times or had been misidentified in the past, an extensive investigation was conducted within the pool of uncommon *Candida* spp. included in the SENTRY global fungal collection (15,271 isolates of *Candida* spp. from four continents) [41]. This study identified a single *C. auris* isolate from Pakistan dating back to 2008, which had not been previously recognized [41]. In 2011, Lee et al. reported the first three cases of bloodstream fungemia caused by *C. auris* highlighting antifungal resistance and the ability to cause invasive infections [42]. One of these cases was incidentally recognized by molecular identification of a microbiological sample obtained in 1996 as invasive fungal infection isolate. To our knowledge, there are no other unidentified *C. auris* strains prior to 1996.

**Fig. 2:**
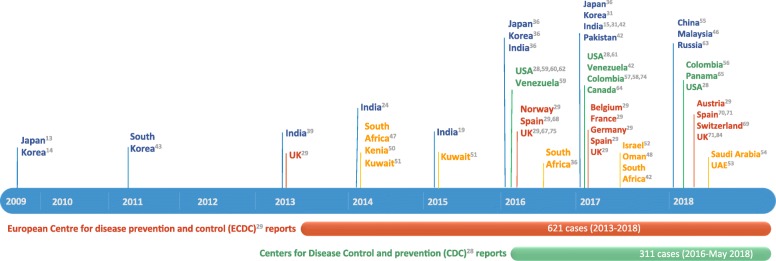
Timeline chart of C. auris reported cases. The reports from the European Centre for Diseases Prevention and Control (ECDC) and the Centers for Disease Control and Prevention are ongoing

**Fig. 3:**
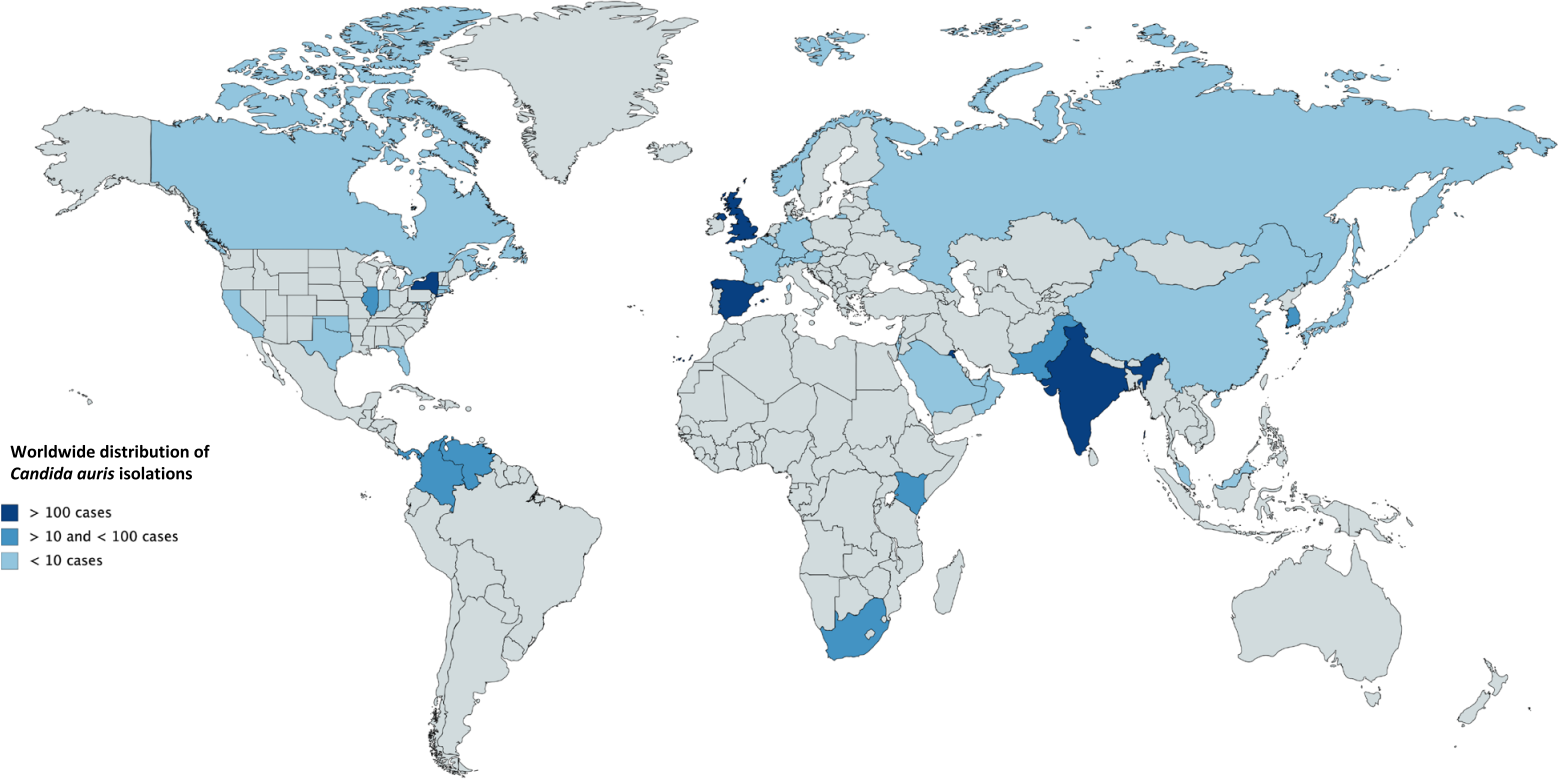
Worldwide distribution of C. auris reported cases

The first “named” description of *C. auris* as a new emergent pathogen has been reported in 2009 by Satoh et al. [13]. The authors reported a single isolate from the discharge of the external ear canal of a 70-year-old inpatient at Tokyo Metropolitan Geriatric Hospital (Tokyo, Japan). Phenotypic, chemotaxonomic, and phylogenetic analyses indicated an affiliation to *Candida* genus, with a close relation to other unusual species [13] such as *C. haemulonii* and *C. pseudohaemulonii*. Later, in South Korea [14], 15 patients affected by chronic otitis media were identified to be infected by unusual and clonally related yeast isolates of *C. auris* confirmed by genomic sequencing [43]. Since the first isolation, *C. auris* infections have been reported from many countries, including India [15, 24, 38, 44], Pakistan [41], South Korea [42], Malaysia [45], South Africa [46], Oman [47, 48], Kenya [49], Kuwait [50], Israel [51], United Arab Emirates [52], Saudi Arabia [53], China [54], Colombia [55–57], Venezuela [58], the United States (US) ((https://www.cdc.gov/fungal/diseases/candidiasis/tracking-c-auris.html); [59–61]), Russia [62], Canada [63], Panama [64, 65], the United Kingdom (UK) [66], and continental Europe [28, 67–70]. Figure 2 shows *C. auris* reported isolations in chronological order. Figure 3 shows the worldwide distribution.

Europe’s burden of *C. auris* outbreaks appears to be increasing, although the epidemiological profile is not completely defined [28]. Recently, the ECDC published a survey on reported cases of *C. auris* and laboratory capacity in Europe, with the purpose to implement surveillance and to control its further spread [28]. Six hundred and twenty cases of *C. auris* were reported in a period from 2013 to 2017, with two countries experiencing four hospital outbreaks. Sporadic cases have been identified since 2013 from different patients throughout England. The first outbreak of *C. auris* in Europe occurred in a London cardio-thoracic center between April 2015 and July 2016; 50 cases were identified, with ability for rapid colonization and transmissibility within the healthcare setting, leading to a serious and prolonged outbreak [66]. The first *C. auris* invasive infection in continental Europe occurred in Spain, where four patients admitted to the surgical intensive care unit of Valencia La Fe University and Polytechnic Hospital (Valencia, Spain) between April and June 2016 were diagnosed with deep-seated infection caused by this “super-fungus” [67]. Despite efforts in limiting diffusion of this pathogen, new colonization cases have continued to appear until now, with a tendency to acquire an endemic pattern. During the study period from April 2016 to January 2017, 140 patients were colonized by *C. auris* and 41 patients underwent candidemia episodes, with 5 patients developing septic metastatic complications. This is the largest ongoing European clonal outbreak [69], involving a different strain from those previously reported, as demonstrated by genotype analysis.

Chowdhary et al. in 2013 were the first to report an outbreak of *C. auris* infection in India, identifying 12 patients with positive microbiological clinical samples collected between 2009 and 2012 [38]. Since then, there has been a progressive increase in the number of clinical cases reported. The high prevalence of invasive infections due to *C. auris* has become a great concern in India, as inter- and intra-hospital spreading of this multi-resistant pathogen has been demonstrated [15]. Public Indian institutions are characterized by higher prevalence of *C. auris* isolation than private hospitals, possibly connected to overcrowding and compromised infection control measures [15], with *C. auris* prevalence ranging from 5 to 30% of all candidemia cases in certain institutions [15, 24, 38, 44]. Recently, *C. auris* was found to be the second most prevalent species causing candidemia in a tertiary care trauma center in Delhi, India, warranting more effective infection control practices to prevent its spread [43]. Moreover, outbreaks of candidemia in Pakistan could be related to the interregional spread of the pathogen, as demonstrated by genomic sequencing of Indian and Pakistani isolates [41].

In US, the Center of Disease Control and Prevention (CDC) issued a clinical alert in June 2016 informing clinicians, laboratories, infection control practitioners, and public health authorities about *C. auris*. It requested that all cases be adequately reported to authorities and to the CDC [71, 72]. This report describes the first seven US cases of *C. auris* infection occurring during May 2013 and August 2016. Six of seven cases were identified through retrospective review of microbiology records from reporting hospitals and reference laboratories [60]. As of May 2018, CDC recorded 311 confirmed and 29 probable cases of *C. auris* infection. Most *C. auris* isolates in the US have been detected in the New York City area and New Jersey (https://www.cdc.gov/fungal/diseases/candidiasis/tracking-c-auris.html). Available epidemiological information suggests that most strains of *C. auris* isolated in the United States were introduced from abroad. Isolates from Illinois and New York were of the same clade as those from South America and South Asia respectively [61]. However, it is not possible to exclude that most of these cases were acquired in the US following local transmission in healthcare settings [60].

Although imported cases have been demonstrated as in US healthcare outbreaks, one of the major questions regarding *C. auris* spread is whether it emerged independently in different countries or if a single outbreak spread from an original source [40]. Using WGS (whole genome sequencing) and SNP (single-nucleotide polymorphism) analysis together with epidemiological observation [25, 41], it was possible to demonstrate an independent and simultaneous emergence of different *C. auris* clonal populations on different geographical areas. Specifically, it was possible to identify the emergence of four different clades (East and South Asian, African, South American) in as many different regions. Transmission within the healthcare setting is suggested by the clonal relatedness of isolates in different institutions [24, 38, 40, 67].

Different reports have been published from South America. The first outbreak was reported in Venezuela between March 2012 and July 2013 [58]. All the isolates were initially identified as *C. haemulonii*. However, isolation of *C. auris* was later confirmed by genome sequencing. The Venezuelan outbreak resulted in *C. auris* being the sixth most common cause of candidemia in the involved institution. In Colombia, sporadic cases have been reported since 2012 [55–57]. Interestingly, an outbreak was reported in a pediatric intensive care unit in 2016, where five cases of invasive infections were identified. Shortly after, nine cases have been isolated in Panama, where patterns of resistance detected by both microdilution method were similar to those observed among *C. auris* isolates in Colombia [65].

In Africa, the first identification of sporadic cases and outbreaks were in South Africa and Kenya. The first four South African cases were isolated in 2012–2013 [46]. Other 10 isolates have been detected, demonstrating a close relation but phylogenetically distinct from Pakistan, India, and Venezuela [41]. Instead, according to other studies, isolates from South Africa had sequence similarity with those from UK [73]. *C. auris* has been considered as the most common pathogen responsible for candidemias in a reference hospital in Kenya, accounting for 45 (38%) episodes over a nearly 3-year period [49].

Only a single report of *C. auris* candidaemia has been published to date in Israel [17]. Collected strains were phylogenetically different from those from East Asia, Africa, and the Middle East, indicating an independent emergence of the pathogen. Infections have been reported in different Gulf States, including Kuwait [50], Oman [47, 48], and United Arab Emirates [52]. Recently, the first three cases in Saudi Arabia have been reported [53].

### Clinical characteristics, risk factors, and outcome

In most cases, clinical presentation is non-specific and it is often difficult to differentiate between other types of systemic infections. Most of the reported cases in the last 5 years were isolated from blood and other deep-seated sites of infection (including invasive devices and catheters tips) [16]. Different clinical conditions including bloodstream infections, urinary tract infection, otitis, surgical wound infections, skin abscesses (related to insertion of the catheter), myocarditis, meningitis, bone infections, and wound infections have been related to *C. auris* [15, 18]. However, isolations from non-sterile body sites such as lungs, urinary tract, skin and soft tissue, and genital apparatus may more likely represent colonization rather than infections [18, 74]. As for other *Candida* spp., the presence of signs and symptoms of infections of the site where *C. auris* has been isolated from can help to differentiate between simple colonization and infection [4]. It is important to identify *C. auris* even from a non-sterile body site because colonization poses the risk of transmission, which requires implementation of infection control precautions [71, 72].

To investigate risk factors associated to *C. auris* infections, Rudramurthy et al. conducted a subgroup analysis and comparison of the clinical manifestations of *C. auris* and non-auris cases in 27 Indian ICUs [15]. In accordance with previous studies, risk factors were not different from those associated with invasive infection due to other *Candida* spp. [22], including prior or continuous exposure to broad-spectrum antibiotics and antifungal agents, diabetes mellitus, abdominal and vascular surgery, presence of central venous catheters, urinary catheterization, post-operative drain placement, chronic kidney disease, chemotherapy, blood transfusions, hemodialysis, total parenteral nutrition, immunosuppressive state [75] and neutropenia [45], and length of ICU stay [15, 18, 76]. The incidence of *C. auris* is significantly higher in patients with primary or acquired altered immune response, secondary to therapeutic management of hematologic malignancies, bone marrow transplantation, and other condition requiring immunosuppressive agents [60]. Interestingly, Azar et al. reported the first case of donor-derived transmission of *C. auris* in a lung transplant patient [75], highlighting several implications on microbiological surveillance before transplants.

The crude in-hospital mortality rate for *C. auris* candidemia is estimated to range from 30 to 72% [16, 26, 41, 44, 69]. Available data suggest that the vast majority of infections affects adults, with a propensity for critically ill patients in intensive care unit (ICU) settings. Pediatric patients have only been reported in Asia and South America [77]. A better outcome was seen in this population [42, 58, 77].

### Infection prevention and control

The progressive increase of outbreaks and sporadic cases of *C. auris* infection emphasize the need for adequate prevention measures. According to reports of recent outbreaks, colonization is difficult to eradicate and it tends to persist for months [66, 69]. Prevention of outbreaks has to be based on the early recognition of sporadical cases, identification of reservoirs and prompt notification. Guidance has been released by international organizations such as Public Health England (PHE-UK) [78], the CDC [79], the ECDC [70], and the Center for Opportunistic Tropical and Hospital Infections (COTHI-South Africa) [80], with recommendations regarding the isolation of patients, contact precautions, and cleaning of equipment and environments in contact with affected patients. Prevention and infection control policies are empirical and mainly based on indications formulated for containment strategies for other multi-drug-resistant pathogens. Table 1 summarizes recommendations by the CDC and the ECDC for prevention and control of *C. auris* transmission.

**Table 1:** Key points for *C. auris* prevention and control by the European Centre for Diseases Prevention and Control (ECDC) and Centers for Disease Control and Prevention (CDC)

ECDC	CDC
Correct identification (MALDI-TOF; DNA sequencing of the D1/D2 domain); Clinicians and microbiologists alertness; Notification and retrospective case-finding	Correct identification (MALDI-TOF; molecular methods) Confirmed isolates of *C. auri*s should be reported to local and state public health officials and to CDC
Good standard infection control measures (including environmental cleaning, reprocessing of medical devices and patient isolation) and prompt notification	Infection control measures: • Placing the patient with *C. auris* in a single-patient room and using contact precautions • Emphasizing adherence to hand hygiene • Cleaning and disinfecting the patient care environment (daily and terminal cleaning) with recommended products • Screening contacts of newly identified case patients to identify *C. auris* colonization
Early identification of carriers by using active surveillance cultures (sites considered for sampling include nose/throat, axilla, groin, rectum, insertion sites of venous catheters; clinical samples such as urine, feces, wound drain fluid, and respiratory specimens)	Screening should be performed to identify colonization among potentially epidemiologically linked patients, including: • Current roommates • Roommates at the current or other facilities in the prior month (even if they have been discharged from the facility) Screening for *C. auris* should be done using a composite swab of the patient’s axilla and groin (sites of consistent colonization). Patients have also been found to be colonized with *C. auris* in nose, external ear canals, oropharynx, urine, wounds, and rectum.
Establish the source of the outbreak (epidemiological investigation, cross-sectional patient screening and environmental sampling); prevention of inter-hospital and cross-border transmission Enhanced control measures to contain outbreaks (such as contact precautions, single room isolation or patient cohorting, and dedicated nursing staff for colonized or infected patients)	All laboratories, especially laboratories serving healthcare facilities where cases of *C. auris* have been detected, should: • Review past microbiology records to identify cases of confirmed or suspected *C. auris* • Conduct prospective surveillance to identify *C. auris* cases in the future • Consider screening close contacts of patients with *C. auris* for presence of colonization
Education and practice audits (for healthcare workers and contacts)	Education of all healthcare personnel, including staff working with environmental cleaning services about *C. auris* and need for appropriate precautions; Monitor adherence to infection control practices
Antifungal stewardship	Antibiotic and antifungal stewardship

Although the exact mode of transmission has to be identified, early evidence suggests that *C. auris* spread is mainly related to exposure to contaminated facilities and transmission from healthcare personnel. Persistent outbreaks have been associated with hand transmission and contamination of surfaces [61, 66, 81, 82]. However, the role of healthcare workers still remains difficult to determine. A recent study sampled patients and their contacts, healthcare workers, and environment in four hospitals in Colombia that had previously reported *C. auris* outbreaks, and found *C. auris* on different objects and facilities, such as bedrails, a bed hand-controller, a mobile phone, and floors. Interestingly, positive samples were collected from surfaces with infrequent patient contact but frequent healthcare workers contact (i.e., chairs, bed trays, and medical equipment), and from surfaces with little to no patient contact and infrequent healthcare workers contact (i.e., closet cabinets, door handles, alcohol gel dispensers) [83]. Thus, once *C. auris* is introduced in the hospital setting, environmental contamination evolves well beyond the patient bedside, resulting in recurrent cases of new colonizations.

*C. auris* is able to survive on a wide range of dry and moist surfaces, including plastic where the pathogen may reside for up to 14 days [84]. *C. auris* seems to be resistant to quaternary compounds disinfectants and cationic surface-active products. Disinfectants with sporicidal activity and hydrogen peroxide-based products are indicated to clean surfaces and healthcare facilities, resulting in highest reduction of *C. auris* colony-forming unit (CFU) [81, 85, 86]. Chlorine-based detergents, ultraviolet light, and hydrogen peroxide vapor demonstrated their efficacy in environmental decontamination procedures after patient discharge [61, 66, 87]. However, persistence of *C. auris* within the hospital environment despite disinfection procedures also suggests an involvement of the interaction between the pathogen and surfaces and the length of exposure to disinfectants [88].

In order to curb transmission, authorities recommend adherence to central and peripheral catheter care bundles, urinary catheter care bundle, and care of tracheostomy sites [78, 79]. If feasible, removal of central catheters or other invasive devices may resolve persistent candidemia and improve clinical outcome [58, 67]. Patients colonized or with proven or suspected *C. auris* infection should be kept in isolation under strict contact precautions until microbiological screening and diagnostic results are available [66]. Incoming patients from institutions where proven *C. auris* isolation has been determined should be screened [78]. Suggested screening sites are groin and axilla, urine, nose and throat, perineal and rectal swab or stool sample. Other high-risk sites may be of consideration, including wounds, cannula entry sites, endotracheal secretions, and drain fluids [70].

### *C. auris* virulence factors

*C. auris* possesses virulence factors, such as germination, adherence, formation of biofilms, and production of phospholipases and proteinases [30]. Table 2 summarizes *C. auris* virulence and resistance factors. Although compared to *C. albicans*, *C. auris* forms significantly reduced biofilms, nevertheless, it has the capacity to form adherent biofilm communities on a range of clinically important substrates. Larkin et al. studied 16 different *C. auris* isolates obtained from patients in Japan, India, South Korea, and Germany and characterized their morphology and virulence factors [30]. *C. auris* produces phospholipase and proteinase in a strain-dependent manner and exhibited a significantly reduced ability to adhere to catheter material as compared to that of *C. albicans*. Further, *C. auris* biofilms were mainly composed of yeast cells adhering to catheter material. In contrast, *C. albicans* showed a highly heterogeneous architecture of biofilms with yeast cells and hyphae embedded within the extracellular matrix [30]. Sherry et al. described the ability of *C. auris* to form antifungal-resistant biofilms, against all three main classes of antifungals [87]. These biofilms were shown to be resistant to chlorhexidine and hydrogen peroxide, displaying a less susceptible phenotype than *C. albicans* and *C. glabrata* [87, 89]. More recently, Kean et al. using a molecular approach investigated the genes that are important in causing the *C. auris* cells to be resistant within the biofilm [89]. Transcriptomic analysis of temporally developing *C. auris* biofilms was shown to exhibit phase- and antifungal class-dependent resistance profiles. Differential expression analysis demonstrated that 791 and 464 genes were upregulated in biofilm formation and planktonic cells, respectively, with a minimum twofold change. Notably, in the intermediate and mature stages of biofilm development, a number of genes encoding efflux pumps were upregulated, including ATP-binding cassette (*ABC*) and major facilitator superfamily (*MFS*) transporter suggesting efflux-mediated resistance in *C. auris* [89]. Previously, Ben-Ami et al. also reported significantly greater ABC-type efflux activity, as evidenced by Rhodamine 6G transport, among *C. auris* than *C. glabrata* isolates suggesting efflux-mediated intrinsic resistance of *C. auris* to azoles [17]. Virulence of *C. auris* and *C. haemulonii* has been recently compared with *C. glabrata* and *C. albicans* in an immunocompetent murine model of invasive infection. In this study, authors reported that virulence in *C. auris* appears to be similar to *C. albicans* and *C. glabrata*, suggesting that common gene sequences could play a pivotal role [23]. The whole genome data of the emerging multidrug resistant species and other related *Candida* revealed that *C. auris* shares some notable expansions of gene family described as related to virulence (including transporters and secreted lipases) in *C. albicans* and related pathogens [31]. The pathogenicity of *C. auris* compared to that of other common pathogenic yeast species in the invertebrate *Galleria mellonella* infection demonstrated strain-specific differences in the behavior of *C. auris* in *G. mellonella*, with the aggregate-forming isolates exhibiting significantly less pathogenicity than their non-aggregating counterparts. Importantly, the non-aggregating isolates exhibited pathogenicity comparable to that of *C. albicans* [29]. Finally, the ability of salt tolerance and cell clumping into large and difficult to disperse aggregates of *C. auris* can contribute to its resistance in the hospital environments. Despite the ability to possess the virulence factors, it is observed that the capacity of *C. auris* to express those is much weaker than that of other *Candida* spp., suggesting that this emerging species is not as virulent as the latter species [30, 87].

**Table 2:** *C. auris* virulence and resistance factors

Virulence genes encoding for: Hemolysin, secreted aspartyl proteinases, secreted lipases, phosphatases, mannosyl transferases, phospholipase, integrins, adhesins, Zn(II) 2 cys 6 transcription factor (strain-specif degree of activity)	
Resistance genes: Azoles resistance Transport proteins and efflux pumps (ATP-binding cassette *ABC*; major facilitator superfamilies *MFS*; upregulation of *CDR1*, *CDR2*, *MDR1*) *ERG 11* mutations (substitutions *Y132F*, *K143R*, and *F126T*) and *ERG 11* upregulation Echinocandin resistance *FKS1/2* (encoding echinocandin drug target 1,3-beta-glucan synthase)	
Adherence to surfaces and plastic materials (e.g., catheters)	
Biofilm formation	
Cellular morphology (aggregating and non-aggregating forms)	
Rudimentary pseudohyphae formation	

### *C. auris* profile of antifungal resistance and their mechanisms

The ability of *C. auris* to develop resistance to multiple commonly used antifungal agents may be responsible for its high rates of mortality [76]. Antifungal susceptibility data published so far points out that some *C. auris* strains exhibit elevated minimum inhibitory concentration (MIC) for three major classes of antifungal drugs, i.e. azoles, polyenes, and echinocandins [41]. Table 3 shows *C. auris* MICs and tentative MICs breakpoint for the most common antifungal drugs.

**Table 3:** Minimum inhibitory concentration (MIC) range and tentative MIC breakpoints of *C. auris* for most common antifungal drugs. Data retrieved by Centers of Disease Control and Prevention (CDC) website—https://www.cdc.gov/fungal/candida-auris/recommendations.html

Drugs	MIC range (mcg/ml)	Tentative MIC breakpoints (mcg/ml)
Triazoles		
Fluconazole	0.12 to > 64	≥ 32
Voriconazole (and other 2° generation azoles)	0.032–16	N/A
Polyenes		
Amphotericine B	0.06–8	≥ 2
Echinocandins		
Anidulafungin	0.015–16	≥ 4
Caspofungin	0.03–16	≥ 2
Micafungin	0.015–8	≥ 4

*C. auris* is frequently resistant to fluconazole although isolates with low MICs against fluconazole (2–8 mg/L) have also been recorded in India and Colombia [57, 83, 90, 91]. Recently, reports have also documented high MICs to amphotericin B, voriconazole, and caspofungin. Antifungal susceptibility testing of 350 isolates of *C. auris* in 10 hospitals in India over an 8-year period showed that 90% of strains were resistant to fluconazole (MIC 32 to ≥ 64 mg/L), 2% to echinocandins (MIC ≥ 8 mg/L), 8% to amphotericin B (MIC ≥ 2 mg/L) and 2.3% to voriconazole (MIC 16 mg/L) [90]. In a recent report of *C. auris* candidemia in a tertiary care trauma center in Delhi, India, 45% of *C. auris* isolates exhibited low MICs of fluconazole [91]. Antifungal susceptibility testing of clinical blood isolates and isolates recovered from environmental and body swabs from hospitals in Colombia revealed that all isolates had low MICs to voriconazole, itraconazole, isavuconazole, and echinocandins [83]. The variable rates of azole resistance in different geographic regions suggest localized evolvement of resistance. Although, data underlying the molecular mechanisms related to resistance to common antifungal drug classes in *C. auris* is scarce, the following update is based on a few recent studies:

- *Azole*

The resistance is mediated by point mutations in the lanosterol 14 α-demethylase (*ERG11*) gene. Substitutions *Y132F*, *K143R*, and *F126L* in the gene were detected. Moreover, *ERG11* gene expression can be increased five- to sevenfold in the presence of fluconazole [90]. This gene, in some strains, can be present in an increased copy number, suggesting that increased copy number may be a mechanism of drug resistance in *C. auris* [91]. Mutations in *ERG11* gene associated with the development of fluconazole resistance in *C. albicans* have been detected in a global collection of 54 *C. auris* isolates including amino-acid substitutions specific with geographic clades: *F126T* with South Africa, *Y132F* with Venezuela, and *Y132F* or *K143F* with India and Pakistan [41]. The *ERG11* sequences of Indian *C. auris* showed amino acid substitutions at position *Y132* and *K143* for strains that were resistant to fluconazole, whereas genotypes without substitution at these positions were observed in isolates with low MICs of fluconazole (MIC 1–2 mg/L) [90]. These results suggest that these substitutions would give a phenotype of fluconazole resistance. Specific *ERG11* substitutions in *C. albicans*, including *F126T*, *Y132F*, and *K143R*, are directly associated with resistance and have been shown to exhibit reduced susceptibilities to azoles upon heterologous expression in *S. cerevisiae* [92, 93].

Other mechanisms of azole resistance have been described in *C. albicans*, including upregulation of *ERG11* and upregulation of drug efflux pumps (e.g., *CDR1*, *CDR2*, *MDR1*) due to gain of function mutations in transcription factors (e.g., *TAC1*, *MRR1*) that induce their expression [94]. The orthologs of transporters from the ATP-binding cassette (*ABC*) and major facilitator superfamily (*MFS*) classes of efflux proteins have been reported in *C. auris*. Further, the overexpression of *CDR* genes members of the *ABC* family and *MDR1* member of the *MFS* transporters has been recorded in *C. auris* isolates. Also, a single copy of the multidrug efflux pump *MDR1* and 5–6 copy numbers of multidrug transporters such *CDR1*, *SNQ2*, and related genes have been identified in *C. auris* using WG sequence data [31], while the *TAC1* transcription factor that regulates expression of *CDR1* and *CDR2* is present in two copies in *C. auris* [31].

b) *Echinocandins*

Main mechanisms of echinocandins resistance are mutations in the *FKS1* gene encoding echinocandin drug target 1,3-beta-glucan synthase. *FKS1* gene analysis using *C. auris*-specific *FKS* primers in 38 Indian *C. auris* isolates showed that four *C. auris* isolates exhibited pan-echinocandin resistance (MICs > 8 mg/L). All four resistant isolates had *S639F* amino acid substitution equivalent to the mutation at position *S645* of the hot-spot 1 of *FKS1*, which is associated with resistance to echinocandins in *C. albicans* [90]. In contrast, in the remaining 34 *C. auris* isolates, wild-type phenotype was observed and the isolates exhibited low echinocandin MICs. Also, a single *C. auris* isolate resistant to both echinocandins and 5-flucytosine obtained from London Cardiothoracic outbreak was investigated for mutation analysis in the later study using *WGS* displayed *SNP*, causing a serine to tyrosine substitution (*S652Y*) in the *FKS1* gene [95]. A recent study highlighted the challenges with the antifungal susceptibility testing of *C. auris* with caspofungin, as *FKS1* wildtype isolates exhibited an Eagle effect (also known as the paradoxical growth effect). Resistance caused by *FKS1 S639F* in *C. auris* was further confirmed in vivo in the mouse model of invasive candidiasis [96]. All isolates were susceptible at a human therapeutic dose of caspofungin, except for those exhibiting the *S639F* aminoacid substitution. This result suggests that isolates demonstrating echinocandin resistance are characterized by mutations in *FKS1* and that routine caspofungin antifungal susceptibility testing by broth microdilution method for *C. auris* isolates should be cautiously applied or even avoided [96]. However, micafungin is the most potent echinocandin in MIC testing and susceptibility testing with micafungin or FKS1 sequence analysis would be better indicators for detection of echinocandin resistance in *C. auris* [96].

c) Amphotericin B

The underlying mechanism of amphotericin B resistance has not been investigated so far in *C. auris*. A recent study by Escandon et al. aimed to describe the overall molecular epidemiology and resistances among Colombian *C. auris* isolates. The authors found that despite *WSG* revealed that isolates are genetically related throughout the country, higher resistance rates to amphotericin B were identified in northern regions if compared to central Colombia. Moreover, resistance to amphotericin B has been found to be significantly associated to four newly identified non-synonymous mutations [83]. Furthermore, reported data on susceptibility tests demonstrated that commercial systems (Vitek AST-YS07) could also detect false elevated MICs of amphotericin B. Thus, a cautious approach is recommended for laboratories to perform antifungal susceptibility testing for this yeast [19].

### Therapy: general concepts and new insights

Echinocandins are the first-line therapy for *C. auris* infection, given resistance to azoles and amphotericine B. As resistance to echinocandins has also been described, patients should undergo close follow-up and microbiological culture-based reassessment to detect therapeutic failure and eventual development of resistances. In cases of unresponsiveness to echinocandins, liposomial amphotericin B (as single or combination therapy with an echinocandin) should be prescribed [60, 61, 67, 75] and consultation with an infectious diseases expert is recommended. Furthermore, MICs of azoles, such as itraconazole, posaconazole, and isavuconazole, are low and these drugs show good in vitro activity, possibly explained by the absence of previous exposure of yeast isolates to these agents, or because of the different structure of the azole-target-protein [41].

Drug associations have already been used with success [60, 67]. Synergistic interactions may have a possible role, as demonstrated for micafungin and voriconazole association [23]. Considering the high prevalence and continuous spread of multi-drug resistant isolates of *C. auris*, there is the need to expand the classes of available antifungals. *SCY-078* showed growth inhibition and anti-biofilm activity against *C. auris* isolates, with activity against echinocandin-resistant strains. Moreover, this drug is not affected by common mutations in protein targets and is orally bioavailable [97]. Recently, Basso et al. described the antifungal properties of θ-defensins, 18-aminoacid macrocyclic peptides with potential applications for therapeutic treatment of systemic MDR infections, representing a template for the future development of new antifungals generation [98]. APX001 is a broad-spectrum antifungal agent for the treatment of invasive fungal infections, including species resistant to other antifungal drug classes, inhibiting an enzyme (*Gwt1*) part of the glycosylphosphatidylinositol (GPI) biosynthesis pathway [99]. Results of a study in a murine model of neutropenic disseminated candidiasis conducted by Zhao et al. may have potential relevance for clinical dose selection and breakpoints identification [100]. CD101 is a novel echinocandin with a prolonged half-life and an improved safety profile, allowing once weekly intravenous administration because of its enhanced pharmacokinetic properties [101]. In a recent study, Berkow et al. demonstrated an encouraging in vitro activity against most *C. auris* isolates, including strains resistant to other echinocandins [101].

## Conclusions

Scientific community and clinicians are facing increasing incidence of antifungal resistance. Non-*albicans Candida* spp. infections are progressively emerging in hospitals and ICUs’ settings. *C. auris* with high mortality rates, multi-drug resistance, environmental resilience, and horizontal transmission has become an issue in clinical practice. *C. auris* MDR strains may continue to emerge independently and simultaneously throughout the world in next few years. High level of knowledge and alertness by physicians and healthcare workers, especially in critical care settings, would help to control the spread and improve diagnostic and therapeutic strategies.

## Additional file

Additional file 1: Flow diagram of the systematic search. (PDF 44 kb)

## Abbreviations

ABC: ATP-binding cassette
AmB: Amphotericin B
BSI: Bloodstream infection
CDC: Center of Disease Control and Prevention
CFU: Colony-forming unit
COTHI-South Africa: Center for Opportunistic Tropical and Hospital Infections
ECDC: European Centre for Disease Prevention and Control
GPI: Glycosylphosphatidylinositol
ICU: Intensive care unit
MALDI-TOF: Matrix-assisted laser desorption/ionization time-of-flight
MDR: Multidrug resistant
MIC: Minimum inhibitory concentration
MRSA: Methicillin-resistant *Staphylococcus aureus*
PHE-UK: Public Health England
UK: United Kingdom
US: United States
