# Supplementary figures and images for: Epidemiology, clinical characteristics, resistance, and treatment of infections by Candida auris
Source: J Intensive Care. 2018 Oct 29;6:69. doi: 10.1186/s40560-018-0342-4 (PMC6206635; doi:10.1186/s40560-018-0342-4)

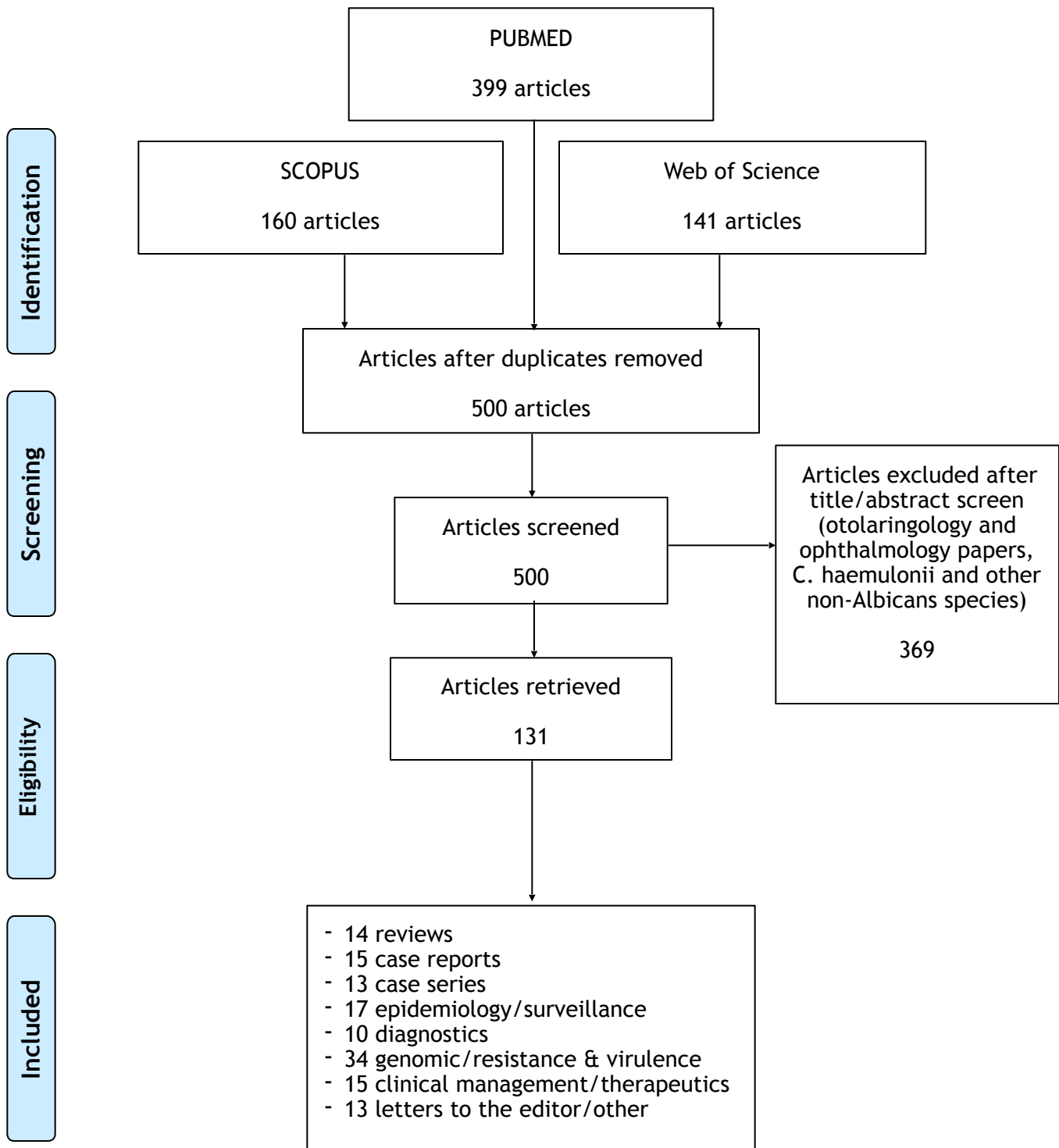

Supplement: Supplementary file 1 — Flow diagram of the systematic search. (PDF 44 kb) [file 40560_2018_342_MOESM1_ESM.pdf]
